# Towards Targeted Delivery Systems: Ligand Conjugation Strategies for mRNA Nanoparticle Tumor Vaccines

**DOI:** 10.1155/2015/680620

**Published:** 2015-12-24

**Authors:** Kyle K. L. Phua

**Affiliations:** Department of Chemical & Biomolecular Engineering, National University of Singapore, 4 Engineering Drive 3, Singapore 117583

## Abstract

The use of nanoparticles encapsulating messenger RNA (mRNA) as a vaccine has recently attracted much attention because of encouraging results achieved in many nonviral genetic antitumor vaccination studies. Notably, in all of these studies, mRNA nanoparticles are passively targeted to dendritic cells (DCs) through careful selection of vaccination sites. Hence, DC-targeted mRNA nanoparticle vaccines may be an imminent next step forward. In this brief report, we will discuss established conjugation strategies that have been successfully applied to both polymeric and liposomal gene delivery systems. We will also briefly describe promising DC surface receptors amenable for targeting mRNA nanoparticles. Practicable conjugation strategies and receptors reviewed in this paper will provide a convenient reference to facilitate future development of targeted mRNA nanoparticle vaccine.

## 1. Introduction

Messenger RNA (mRNA) has achieved great success in an increasing number of biological applications. Apropos, the notion of nonviral genetic vaccination is also increasingly associated with mRNA instead of DNA. Given a mature drug and gene delivery field, mRNA nanoparticle delivery science is often deferred or closely compared with DNA and siRNA systems [1, 2]. However, as various reports have shown, unique properties of mRNA delivery exist [3, 4] and continue to be a relevant research focus today. mRNA delivery science has made significant progress since the first demonstration of cell based mRNA tumor vaccine delivery via RNA loaded DCs [5]. They include the optimization of the mRNA molecular structure [6, 7], direct* in vivo* administration of mRNA [8, 9], delivery routes [3, 4], evaluation of rationally designed gene carriers [10–14], and, recently, self-replicating RNA [15].

Along this developmental trajectory, DC-targeted nanoparticle gene delivery systems may be an imminent next step forward for nonviral tumor vaccine delivery. In this brief report, established conjugation strategies for both polymeric and liposomal gene delivery systems will be described. This will be followed by a brief discussion on three promising DC receptors that are suitable for targeted delivery of mRNA nanoparticles for tumor vaccination.

## 2. Ligand Conjugation Strategies for Gene Delivery Systems

Ligands targeting surface receptors on DCs are molecules grafted onto surfaces of formulated nanoparticles, recognizable by DC-specific uptake mechanisms, and endow nanoparticles with the ability to be taken up exclusively by them. This has the benefit of reducing effective doses of vaccine required through nonspecific uptake by other cell types. In the case of vaccines, which typically contains proinflammatory adjuvant molecules, a decreased dose also has the benefit of reducing undesired side effects. Since a wide variety of nanoparticle delivery systems exist, different ligand conjugation strategies have been developed. In this section, we will discuss three conjugation strategies that are most often applied to gene delivery systems.

**Figure 1:**
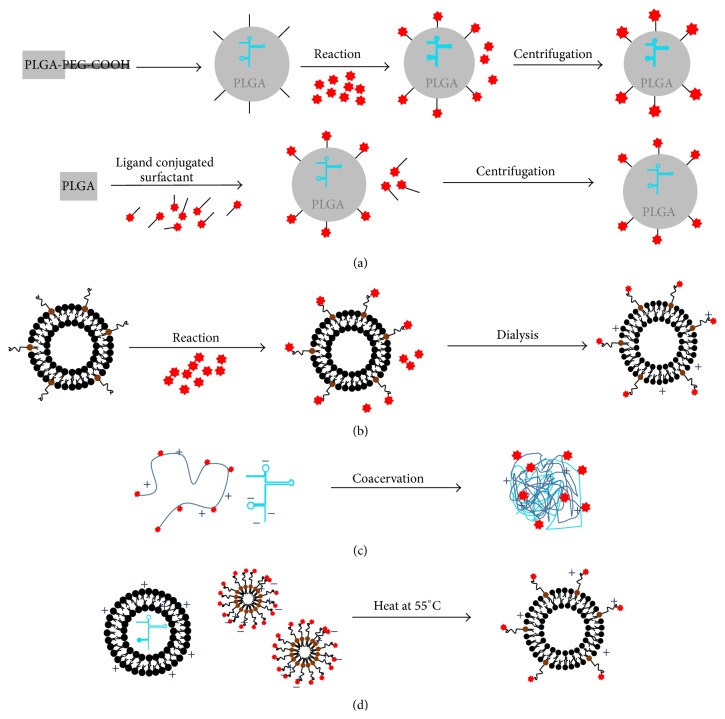
Established strategies for the conjugation of ligands onto polymeric and liposomal nanoparticles. (a) (Top) PLGA (poly(lactic-co-glycolic acid)) nanoparticles formed by copolymer PLGA-PEG-COOH are stabilized with normal surfactant and subsequently reacted with ligands bearing compatible linking groups. (Bottom) PLGA nanoparticles are stabilized with amphiphilic surfactants containing functionalizable molecules. PLGA nanoparticles, susceptible to hydrolysis, are purified by centrifugation to reduce water exposure time. (b) DC-targeting antibodies bearing compatible cross-linkers (e.g., -SH) are reacted with preformed liposomes to form immunoliposomes, which are purified by dialysis. (c) Electrostatically neutral ligands (mannose) are covalently conjugated to cationic polymers and directly used to formulate targeted nanoparticles. (d) Postinsertion functionalization of liposomes/lipopolyplexes. Formulated liposomes/lipopolyplexes are heated with micelles bearing targeting ligands at 55°C for at least 15 mins. The resultant ligand conjugated liposomes/lipopolyplexes can be used without further purification.

First, nanoparticles with solid cores such as poly(lactic-co-glycolic acid) (PLGA) and inorganic nanoparticles (e.g., gold nanospheres, calcium phosphate) possess excellent colloidal stability such that ligands can be covalently conjugated directly onto particles surfaces without aggregation. In PLGA systems, nanoparticles are formulated by emulsion techniques [16–18] using PLGA-PEG-COOH copolymer, which can be synthesized by grafting PEG-COOH onto the ends of PLGA [19]. The resultant mRNA infused PLGA nanoparticles bearing surface carboxylate groups (COOH) can be further functionalized with any ligands bearing amine groups (e.g., peptides, antibodies, nanobodies, and aptamers) via N-hydroxysuccinimide (NHS) chemistry, which proceeds with good efficiencies under physiological conditions if NHS bearing ligands are applied in excess [20] (Figure 1(a), top). However, this conjugation strategy will require the colloidal nanoparticles to remain stable through every step of the conjugation process (surface chemistry modifications, purification and lyophilization). Ligand conjugated nanoparticles are normally purified from the reaction mixture via centrifugation, and hence this strategy is compatible with formulations bearing a solid core because they can withstand compression without aggregation. Apart from centrifugation, dialysis is another common technique used to remove unconjugated ligands. However, dialysis is not compatible with PLGA (as well as other polyesters, e.g., poly-*β*-amino esters) as ester bonds in these polyesters undergo hydrolysis. Conversely, formulations that are chemically inert (e.g., gold nanoparticles, immunoliposomes, and polyamide-based nanoparticles) but aggregate upon centrifugation can be purified by dialysis (Figure 1(b)). A similar approach uses functionalized amphiphilic surfactants commonly used to stabilize the PLGA nanoparticles in colloidal suspension (Figure 1(a), bottom). These surfactants, which bear reactive chemical moieties (e.g., COOH, NH_2_, and OH), are optimally incorporated on particle surfaces and amenable for subsequent conjugation with targeting ligands bearing compatible linkers [21]. In particular, avidin-fatty acid surfactants have been applied to stabilize PLGA nanoparticles [22, 23]. The resulting nanoparticles can be subsequently functionalized with biotinylated ligands such as antibodies, which are easily available, to render user defined DC surface receptor targets such as DEC-205 and DC-SIGN [22, 24, 25]. This formulation is relatively attractive because DC receptors are very often targeted by antibodies. However, notwithstanding the immunological consequences of antibodies, the sheer size of antibodies may result in low surface coverage due to steric hindrance. This can be mitigated with more advanced ligands such as single chain fragment variable (scFv) [26, 27] or aptamers [28], making this an attractive conjugation method.

Second, targeting moieties can instead be incorporated as part of the carrier molecule (polymer or lipid). The ligand conjugated carrier is directly used to formulate the nanoparticles via coacervation between positively charged gene carriers and negatively charged mRNA, and hence no additional step is needed to affix the ligands. This strategy is typically applicable for electrostatically neutral, low molecular weight ligands to ensure that they do not interfere with the carrier molecule during nanoparticle formulation (Figure 1(c)). Mannan/Mannose, a sugar that interacts with C-type lectin/lectin-like receptors, is the most commonly applied DC-targeting ligand incorporated into nanoparticles using this approach. A large number of mannosylated lipids and polymers have been developed hitherto for the purpose of vaccination [29–36]. For liposomal systems, mannose are grafted onto the head groups of lipids [29–31], while, for polymeric systems, they are normally covalently attached along the backbone of polymeric carriers [32–36]. Most of these systems are tested for delivery of different vaccine molecules including peptides, DNA, and siRNA with a consistent improvement in uptake efficiencies over nonmannosylated nanoparticles, which translates to an improved immunization outcome. Notably, Midoux group elegantly demonstrated, as a proof-of-concept, that mRNA-loaded mannosylated lipophosphoramides target DCs* in vivo* and translate into a better survival outcome based on a B16-F10 prophylactic tumor model [31, 37].

Third, another tried and tested strategy for ligand conjugation primarily in liposomal systems exploits the use of hydrophobic interaction (Figure 1(d)). It is well known that liposomes/lipopolyplexes are not thermodynamically stable colloids that aggregate slowly over time [38–40]. Aggregation is a fusion process when hydrophobic interactions between the lipid tails are stronger than the repulsive forces on the surfaces of the liposomes. Factors determining this balance include temperature, ionic concentration of the buffer, and amphiphilic property (surface charge of the lipids versus length and number of the lipid tails). Exploiting effects of temperature on lipid fusion, liposomes or lipopolyplexes encapsulated with mRNA or other payloads can be incubated with ligand-micelles (e.g., DSPE-PEG-2000-X, where X = ligand) at a temperature of 55°C for at least 15 minutes. Due to increased hydrophobic interaction at a higher temperature, ligand conjugated lipids from these micelles can be transferred to the liposomes, effectively decorating them with the desired targeting ligands. These ligand conjugated micelles can be prepared by reacting thiol (SH-) or amine (NH_2_-) bearing ligands with DSPE-PEG-NHS or DSPE-PEG-maleimide (DSPE: 1,2-distearoyl-sn-glycero-3-phosphoethanolamine) available commercially with different PEG molecular weight. This so-called “postinsertion” strategy is a facile approach to functionalizing liposomes with any desired ligands. Unlike PLGA system, DSPE-PEG-ligand can be prepared separately and conveniently incorporated into formulated liposomes on demand [41–43]. The amount of PEG coverage over a 100 nm liposome needed to prevent aggregation in serum is determined to be >8 mole% (based on total lipid content) in the liposome formulation [44, 45]. A caveat to postinsertion strategy is that if the amphiphilicity of the micelles is significantly affected by an excessively hydrophilic head (e.g., highly charged aptamer, long PEG chain), postinsertion method may fail because the increased hydrophobic interaction induced at a higher temperature may not be sufficient to trigger micelle fusion with the liposomes/lipopolyplexes.

## 3. Targeting mRNA Nanoparticles via Selective Endocytic Pathways

When particles are administered into the body, unless the injected site is already the lymph node (e.g., intranodal administration) or has a high density of antigen presenting cells (e.g., intradermal or intranasal administration), nanoparticles need to be passively transported from the site of administration to the lymph nodes via the body's circulatory system such as the lymphatics or the systemic circulation [28, 46–48]. During passive transport from the site administration to the lymphoid tissues, nanoparticles may be taken up nonspecifically by bystander cells based on a range of physiochemical factors such as size, surface charge, and chemical structure of surface molecules. Targeting ligands may reduce such occurrences due to incompatible surface chemistries while increasing uptake efficiencies of nanoparticles when reaching the target site [49–51].

There are different interpretations of “targeted delivery.” While generally it means selective delivery of the vaccine to DCs bearing specific surface receptors, direct outcome of receptor binding depends on what receptors are being targeted. Targeting ligands can, amongst other functions, help increase the uptake by binding to receptors designed to endocytose larger particles [50], mitigate repulsive forces [51], or improve surface compatibility between the particles and the cell membrane [49]. Since intracellular fate of the particles taken up by endocytosis [52] is determined largely by the mechanism through which they are being taken up, targeting ligands may help direct endosomes into specific intracellular trafficking pathways that are less degradative so that gene delivery efficiencies are increased.

DCs, unlike other somatic cells, possess unique endocytic receptors catered to antigen uptake and processing. These receptors are special because they not only trigger particle uptake, but also mediate cross presentation and the development of the immune response. Although cross presentation in DCs influenced the development of subunit nanoparticle vaccines, its impact on genetic vaccination is less conclusive.

**Figure 2:**
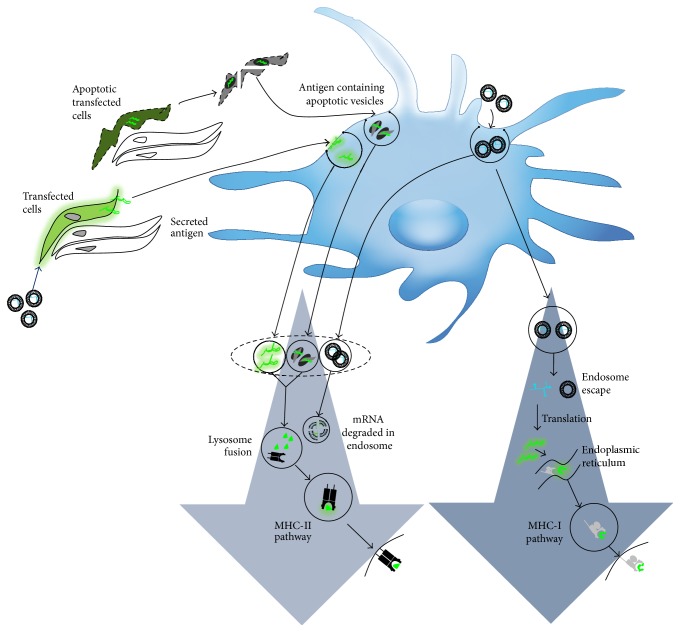
The genetic vaccination model. Antigen presentation occurs directly by transfected DCs through gene expression of the antigen. DCs also cross present antigens secreted by transfected bystander cells, or derived from phagocytosis of apoptotic cells. Cross presentation mechanisms in DCs may facilitate delayed lysosomal delivery leading to higher delivery efficiencies.

The genetic vaccination delivery model has been described as a process where both bystander and antigen presenting cells are transfected [53, 54]. According to this model, as illustrated in Figure 2, antigen presentation occurs through direct transfection of DCs and also through indirect transfer by transfected bystander cells. When the mRNA nanoparticles are targeted to DCs directly, those that escape the endosomes will have a higher chance of being expressed. In DCs, endosome escape not only depends on the efficiency of the gene carrier, but also depends on the trafficking mechanisms. For example, cross presentation mechanisms in DCs can disrupt lysosome trafficking pathways via mediation of endosomal pH leading to higher delivery efficiencies [55]. But, on the other hand, intracellular trafficking pathways of nonprofessional antigen presenting cells often terminate at the lysosomes. When mRNA nanoparticles are delivered without specific DC-targeting ligands, they will also transfect bystander cells. The latter provide an alternative source of antigens by secreting them (if the antigens are secretory in nature or designed with a secretory signal) into the extracellular space for capture by DCs. Finally, according to the consensus genetic vaccination, the other indirect delivery mechanism occurs when transfected bystander cells become apoptotic due to significant stress caused by viral or tumor infection. DCs then acquire antigen through phagocytosis of these apoptotic cells.

Sufficient literature exists to suggest that indirect delivery mechanisms via bystander cells does not play a significant role in targeted delivery systems since targeted genetic nanoparticle vaccines consistently improve immunization outcomes [32–34, 56–58]. DC-specific receptors that not only increase uptake but also enhance transfection via less degradative intracellular trafficking pathways will be attractive for mRNA nanoparticle tumor vaccination [59]. While a long list of DC receptors has been discovered to possess immune modulating function, only a few may benefit mRNA delivery beyond uptake enhancements because they are also targeted towards less degradative intracellular trafficking pathways [60]. They are type I C-type lectins such as CD205 (DEC-205) and CD206 (macrophage mannose receptor) and type II C-type lectins such as CD370 (CLEC9A/DNGR-1). These will be briefly described.

### 3.1. DEC-205

DEC-205 is ubiquitous receptor found on almost every conventional dendritic cell [61]. It is a type I C-type lectin-like molecule consisting of a single polypeptide chain that functions as recycling endocytic receptor and caters for a wide range of cargos that include, notwithstanding lectin-like molecules, apoptotic cells [62], necrotic cells [63], and CpG [64]. DEC-205 is an attractive target receptor because antigens delivered via this receptor are presented on both MHC-I and MHC-II molecules [63]. Furthermore, engagement of DEC-205 does not lead to proinflammatory response, making it an attractive receptor target for tolerance immunization [65]. The anti-DEC-205 ligand is one of the most developed ligands in immunotherapy. While ligands targeting most of the other DC-specific receptors continue to manifest in antibody molecules, anti-DEC-205 ligands in form of scFv [26, 27] and aptamer [66] have been reported.

Functional properties of DEC-205 will benefit mRNA vaccination via higher transfection efficiencies. For example, being a cognate endocytic receptor for apoptotic cells, DEC-205 will efficiently uptake both nano- and microparticles it comes into close contact with. Hence, given mRNA nanoparticles tendency to aggregate* in vivo* (increased particle sizes), administered dose will have higher bioavailability when targeted towards DEC-205. In addition, cross presenting properties of DEC-205, thought to be results of “leaky endosomes” or less degradative endocytic pathway, will facilitate endosome escape of mRNA nanoparticles into the cytoplasm and avoid the lysosomes.

### 3.2. Mannose Receptor

The mannose receptor, another type I C-type lectin receptor with a well-established role in tissue homeostasis [67], recognizes sulfated carbohydrates, collagen, and oligosaccharides through its cysteine-rich domain [68, 69], fibronectin domain [70], and C-type lectin domains [71, 72], respectively. The mannose receptors have been well-known endocytic receptors for decades in part because they are extensively studied as scavenging receptors in macrophages, which were initially thought to be the major antigen presenting cells before DCs were discovered. The ligand for this receptor is mannose residue grafted on the gene carrier [29–36] as previously described. Its role in antigen presentation was conclusively determined through the use of DCs derived from mannose receptor negative transgenic mice [73]. This study confirmed that DCs' mannose receptors not only serve as uptake receptors [74–77], but also mediate cross presentation of soluble mannosylated antigens [78–80]. Since payload taken up via mannose receptor stably accumulates in the early endosome and is excluded from lysosomes for up to 6 hours [78, 79], this intracellular trafficking pathway is expected to be less degradative and highly attractive for mRNA nanoparticle delivery.

### 3.3. CLEC9A/DNGR-1

CLEC9A (C-type lectin domain family 9, a.k.a. DNGR-1 or CD370) is a recently discovered endocytic receptor that is implicated in the clearance of damaged [81] and dead [82, 83] cells. This receptor, currently targeted via antibody, is restricted to a very small population of blood BDCA3^+^ DCs [84] (in humans) and its equivalent in mice models is CD8^+^ DCs. Due to its endocytic nature, antigen delivery properties of CLEC9A are rapidly investigated [85, 86]. Recent reports show that CLEC9A are effective in cross presenting antigens for cell mediated immunity [83, 87] and can be as effective T cell activators compared to Langerin and DEC-205 [88]. Similar to other receptors capable of cross presenting soluble antigens, nanoparticles targeted to CLEC9A are expected to enter a less degradative intracellular trafficking pathway, leading to higher transfection efficiency. Restricted expression of CLEC9A to blood DCs may limit it as a practical receptor compared to the mannose receptor and DEC-205 for targeted delivery to conventional DCs. Nevertheless, CLEC9A remains an attractive receptor for targeting plasmacytoid DCs [86].

## 4. Conclusion

As a late bloomer, development of mRNA therapeutics benefits from a plethora of related knowledge on similar delivery systems. Advancing from passive targeting strategies employed for most mRNA nanoparticle tumor vaccine to date, active targeting of mRNA nanoparticles to DCs will further improve current therapeutic outcome for the treatment of cancer. Practicable conjugation strategies as well as target receptors reviewed in this paper will provide a convenient reference to facilitate future development of targeted mRNA nanoparticle vaccine.
